# Making the equivocal unequivocal: standardization of clean margins in diabetic foot osteomyelitis

**DOI:** 10.1186/s40842-020-00096-2

**Published:** 2020-05-20

**Authors:** Brian M. Schmidt, Christine Jarocki

**Affiliations:** Michigan Medicine, Department of Internal Medicine, Division of Metabolism, Endocrinology, and Diabetes, 24 Frank Lloyd Wright Drive, Lobby C, Ann Arbor, MI 48106 USA

**Keywords:** Amputation, Bone histopathology, Diabetic foot ulcer, Residual bone culture, Osteomyelitis

## Abstract

**Background:**

The prevalence of diabetes mellitus continues to rise. Diabetic foot ulcers with osteomyelitis are a diabetes-related complication presenting a significant burden to this cohort. A cure to diabetic foot osteomyelitis remains elusive and standard of care has failed to improve outcomes. To advance research and better patient outcomes, the authors offer specific guidance with terminology to enhance operative dictations which may improve surgical practice and guide treatment.

**Methods:**

A consecutive review of podiatric surgical dictations for inpatient diabetic foot osteomyelitis within a tertiary care facility was performed. Surgical descriptors of bone were standardized: density, anatomic structure, vascular thrombosis, color, and draining sinus. Correlations between the five categories and histopathological results were performed after kappa analysis for interrater reliability was performed.

**Results:**

Kappa coefficient demonstrated high inter-reliability of surgical findings. This suggests potential agreement amongst surgeons performing similar procedures. It was also found that specific bone descriptors had moderate to strong correlation with clean histopathologic bone margins when biopsied. This further suggests that the use of standardized terms may help guide definitive therapy.

**Conclusions:**

The authors suggest a standardized approach which includes consistent descriptors of intraoperative bone. With use of standardized terms, vague and blanket descriptors are eliminated. This has potential to improve understanding of changes within bone as a result of infection and diabetes. Early and improved communication of intraoperative findings will enhance the multidisciplinary approach. This could potentially lead to changes in diabetic foot management and may limit hospital waste waiting for final cultures and pathology reports.

## Background

It is now estimated 34.2 million people in the United States have Diabetes Mellitus (DM) [1]. Approximately 15–25% of diabetics will develop a diabetic foot ulcer (DFU) [2]. Further estimations suggest between 40 and 80% of DFU become infected [3]. Infections vary in severity, but approximately 20% of DFU with soft tissue infection progress to diabetic foot osteomyelitis (DFO) [4]. The 2012 Infectious Diseases Society of America [IDSA] Diabetic Foot Infection Classification system and Clinical Practice Guideline is a common reference used by physicians to determine treatment options [5]. Correlations with the IDSA classification system suggest 10–15% of *moderate* infections proceed to DFO, while 50% of *severe* infections proceed to DFO (Table 1) [5, 6].

**Table 1:** Adapted from IDSA Diabetic foot infection classification system [5]

Manifestation of infection	IDSA infection severity
No symptoms or signs of infection *Recall, infection is when 2 or more of the following are present: erythema, edema, pain, warmth, purulence*	Uninfected
Local infection within the skin or subcutaneous tissue, and where erythema is restricted to > 0.5 cm to ≤2 cm surrounding the ulcer, No systemic signs of infection	Mild
Local infection as above, with erythema > 2 cm, or deeper tissues affected, No systemic signs of infection	Moderate
Local infection as above with systemic signs of infection	
*Recall, systemic signs include 2 or more of the following:*	Severe
*Temperature > 38 °C or < 36 °C*	
*Heart Rate > 90 beats/minute*	
*Respiratory Rate > 20 breaths/min or PaCO*_*2*_ *< 32 mmHg*	
*White Blood Cells > 12,000 or < 4000 cells/*μL *or 10% immature bands*	

Diagnosing DFO is difficult, but consensus indicates diagnosis can be determined by three methods: visualizing exposed trabecular bone within an ulcer, magnetic resonance imaging (MRI), or biopsy of bone for microbiology and/or histopathology [7]. Having exposed trabecular bone indicates that the cortex has been compromised. Advanced imaging with MRI is best for diagnosing osteomyelitis because of its ability to display anatomical detail and it has a high sensitivity for detecting early infection [8, 9]. Obtaining a bone sample is historically the gold standard for diagnosis [10, 11], but it does come with challenges to its legitimacy [12–15].

There are obvious benefits to obtaining a bone sample. Osseous samples are collected for microbiologic exam to identify a causative pathogen. Pathogen identification is used to guide definitive (narrowed) antimicrobial therapy [5]. Osseous samples are also examined for a histopathologic exam to visualize bone cell death, acute or chronic inflammation, and reparative response [16]. When a standardized process is used for histopathologic evaluation of suspected osteomyelitis, pathologists demonstrated high inter-reliability [17]. Furthermore, patients managed based off these standardized histopathology results were prospectively found to have reduced readmission rates and reduced negative outcomes, especially when bone biopsied from the residual bone demonstrated no osteomyelitis [17].

Obtaining bone biopsies from suspected clean residual bone is not yet a standardized practice amongst surgeons, though is a recommended [5, 7]. A retrospective report reviewing surgical practice from 132 lower extremity amputations suggested that culturing clean residual bone is highly dependent on surgeon preference. The authors annotate the reality of the operating room, in that it is up to the surgeons to define the margin between dirty and clean bone [18]. Clinical appearance of bone, coupled with perioperative imaging, guides surgical resection; however, there are no direct guidelines with how much bone to resect or what constitutes non-infected bone [5, 7].

There is continual controversy as to whether antimicrobial therapy or bone resection is the best treatment option for DFO [5, 16, 19, 20]. In fact, the argument between medical and surgical management may only be an easy choice when given clinical extremes, with minimal or massive bone involvement [5, 7]. When surgical resection is performed, it is recommended to be completed by an experienced surgeon [5, 19]. However, there is no defined qualification to determine an experienced surgeon. Furthermore, “best practice guidelines” are lacking in surgical management for DFO [7].

Given this, we reviewed surgical descriptors of dirty and clean bone in operative reports. We first assessed if surgeons were examining bone, presuming this would be reflected in surgical findings. Examining dirty, infected bone confirms the diagnosis of osteomyelitis. Moreover, examining infected bone and uninfected bone, determines the margin in which a surgeon performs the resection. We then reviewed how surgeons dictated their surgical findings, to determine if there were specific patterns with terminology. Finally, we correlated terminology with histopathology results. We also provide evidence which supports continued excision of bone until surgeon descriptions match that of clean, uninfected bone.

## Methods

We designed a retrospective consecutive review of 50 patients with DM who underwent ablative surgery for suspected DFO during their admission at a large tertiary academic hospital. Surgeries were performed between 2015 and 2018, by five faculty podiatrists.

Patients with diabetic foot infections [DFI] were managed according to the institutional guidelines developed by an interdisciplinary team to coordinate appropriate care. The guidelines establish key points in diagnosis and treatment of DFI with an algorithm for when to consult different specialties. Diabetic foot osteomyelitis was diagnosed preoperatively from a combination of physical exam, laboratory values, and advanced imaging studies. All patients were medically optimized prior to surgical intervention. If patients did not have palpable posterior tibialis and dorsalis pedis pulses, peripheral arterial disease was evaluated with non-invasive Doppler ultrasound to obtain ankle-brachial and toe-brachial indices. Adequate pedal perfusion is defined by an absolute toe pressure above 40 mmHg and this is confirmed prior to surgical management.

Procedures included within the study were digital, ray or transmetatarsal amputations, and incision and drainage of infected feet, under local anesthesia and monitored anesthesia care. Standardized practice within podiatry at the institution consist of sending suspected clean, residual bone margins for microbiology and histopathology [17, 21]. First, the primary amputation or resection was completed. After all nonviable tissue was removed, the surgical site was irrigated with copious amounts of sterile normal saline. The surgeons donned new gloves and using new sterile instruments, specimens were separated to obtain clean bone margins. Specimens were sent for microbiology, to assess aerobic, anaerobic, acid-fast, and fungal growth cultures. Histopathology was sought to determine cellular structure.

An independent third party randomly assembled 50 podiatry dictations for surgeries that were coded with osteomyelitis in the preoperative and postoperative diagnosis. Two surgeons separately reviewed the brief-operative notes and operative notes for these random charts and coded the descriptors of bone accordingly using standardized nomenclature. They assessed words used to illustrate “dirty” and “clean” bone within the surgical findings.

Five categorizes were agreed upon to classify bone descriptor words: density, anatomic structure, vascular thrombosis, color, and draining sinus. The specific categories were chosen using a combination of classification features and radiographical and advanced imaging finds for osteomyelitis. The most common etiology for DFO, with the presence of a DFU, is contiguous spread. Pathogens penetrate the cortex prior to invading the bone marrow [19]. Bacteria can additionally induce osteolysis and hide intracellularly [22]. Thus, it would be expected that the density of infected bone would be softer. There would also be anatomical abnormalities, such as erosions or pathological fractures. Infection can occlude vascular infiltration, which may exhibit as vascular thrombosis [22]. Color was chosen as a category given the simplicity. Infected bone or necrotic bone, would not display a white appearance as uninfected living bone would. Draining sinus was included to account for any “pus in bone” that is considered a clinical sign of osteomyelitis [9, 19]. The bone descriptors used in the dictation were determined to fall into these categories. If a description did not fit within a category, it was disregarded.

Correlation coefficient, r, was then drawn between each bone descriptors for the “clean” bone and final histopathological reports. Further correlations were drawn between a combination of descriptors and final histopathological reports.

## Results

Kappa coefficient is a statistical measure of interrater reliability. It was used here to determine if the authors agreed with categorizing the dictation findings. The authors demonstrated high inter-rater reliability, with near perfect kappa coefficient for assessment of both dirty and clean bone descriptors (Table 2).

**Table 2:** Kappa coefficient values demonstrating interrater reliability for dirty bone and clean bone descriptors, respectively

**Dirty Bone**		
**Descriptor**	**Kappa Value**	**Agreement**
Density	0.96	Excellent
Anatomic Structure	1	Perfect
Vascular Thrombosis	0.72	Moderate
Color	1	Perfect
Draining Sinus	1	Perfect
**Clean Bone**		
**Descriptor**	**Kappa Value**	**Agreement**
Density	1	Perfect
Anatomic Structure	0.72	Moderate
Vascular Thrombosis	0.83	Excellent
Color	0.96	Excellent
Draining Sinus	1	Perfect

Specific terms were commonly used. Words that were considered to describe bone density included “soft” and “hard”. Anatomic structure was considered to be described by words like “erosions”, “contour”, and “caliber”. Vascular thrombosis terms consisted of “necrosis” or “avascular necrosis”, while color was described by direct terms such as “black”, “yellow”, “tan”, or “white”. Lastly, draining sinus was included to account for any “pus in (medullary) bone”.

Several patterns were revealed within the dictation review. Density descriptors were the most commonly used terms to describe “dirty” bone and was used in 39/50 dictations. Meanwhile color change was not used at all. However, in describing “clean” bone, both density and color were routinely documented in 45 and 41 of the reviewed dictations, respectively.

Furthermore, among the dictations, it was commonly seen that soft tissue was more emphasized than bone. Blanket terms were also used to describe the entire surgical site: “healthy tissue” “viable” “nonviable”; present in 30% of dictations. This drew attention in that much of these descriptors are left to interpretation by the reader, rather than being definitive terms describing what is visualized to the reader.

The correlation coefficient between each descriptor for suspected “clean” margins and confirmed clean histopathological proximal bone was also moderate-to-strong (Table 3). There was a relatively weak association found using a single bone descriptor. However, the association increased when combining two bone descriptors.

**Table 3:** The correlation coefficient between each descriptor for suspected “clean” margins and confirmed clean histopathological proximal bone

Clean Bone			
Descriptor	R Value	Association	*p* value
Anatomic Structure	0.03	None-to-weak	> 0.05
Density	0.09	Weak	< 0.01*
Vascular Thrombosis	0.15	Weak	< 0.01*
Draining Sinus	0.2	Weak	> 0.05
Color	0.35	Weak-Moderate	< 0.001*
Density + Bleeding or Color	0.65	Moderate-Strong	< 0.01*

* indicates a significant *p*-value

## Discussion

Prevalence of DM continues to increase [1]. Given that a common complication of DM is a DFU with potential DFO, reviews of “best practices” ensure patients are appropriately managed. Multiple reports document that poor clinical outcomes are associated with residual osteomyelitis from surgical resection [21, 23]. Direct visualization of the bone from the surgeon appears to be the limiting factor in surgical resection. By standardizing terms used to describe the bone in surgical dictations, it may increase the prevalence of obtaining clean residual margins, which would improve patient outcomes as described by previous literature [17, 21].

It is not clear at this time why the correlation coefficients may not be as clinically meaningful with the reflected values. One explanation is the current lack of surgical standardization in DFO management, which our study is the first to identify. By implementing a new taxonomy to improve surgeon characterization and description of bone, the authors anticipate stronger correlation values with additional prospective research. This knowledge will enhance, in real-time, surgical management of DFO and alter current practices. A surgeon may decide to resect bone based on the proposed descriptors, rather than preference. Furthermore, a surgeon may resect bone until multiple findings are met, knowing that multiple descriptors are more supportive with clean margins.

Another potential enhancement from creating this “best practice” would be to improve communication amongst the multidisciplinary team once integrated into standard work flow. A multidisciplinary team is quickly becoming the standardized method to treat diabetic foot infections [24, 25]. Podiatrists work alongside vascular surgeons, infectious disease consultants, internal medicine, and other specialties. By improving surgical dictations and documentation, we have the ability to the improve direct communication. Standardized terms remove a majority of the subjective component that is not translated through with blanket terms, such as “viable” and “nonviable”. The standardized template includes the five categories of bone descriptors, with recommended terminology (Table 4).

**Table 4:** The standardized surgical dictation with suggested terminology

Descriptor	Terminology
*Density*	Hard, Soft
*Anatomic Structure*	Erosion, Cortical integrity, Fracture, Bone Marrow infiltration, Caliber, Cartilage
*Vascular Thrombosis*	Paprika Sign, Presence of clot
*Color*	White, Yellow, Black, Gray
*Draining Sinus*	Pus in bone

The authors acknowledge there are limitations to the proposed standardized terminology. The current review reflects a small sample of podiatrists within one institution, which may not accurately reflect how dictations are performed more broadly. To be considered for “best practice”, a larger sample size among multiple institutions, and with a variety of surgeons, including plastics, orthopedics, and vascular specialties, would improve the understanding of how surgeons describe bone. From there a set list of standardized terms could be completed. Correlations should be greatly enhanced by understanding which terms may be positively associated with clean residual margins. Collaboration within a multidisciplinary team to define categories would also improve this classification. Infectious disease, endocrinologists, and pathologist, amongst others, may have unique insight with how dirty, infected bone may present and how clean, uninfected may look and feel.

## Conclusion

Obtaining clean residual bone margins is an important prognostic factor for patients with DFO. Surgeons should collect these samples intraoperatively. Furthermore, they should precisely and accurately document the surgical dictations in detail (Table 4). In DFO, standardizing descriptors of bone viability for surgical resection would assist surgeons by more accurately informing the surgeon of non-infected versus infected bone at the point of care (i.e. in the operating room). Detailed surgical documentation may also improve communication amongst the multidisciplinary team. This has the opportunity to guide patient therapy. We believe this enhancement with practice will have improved outcomes for patients.

## Data Availability

Not applicable.

## Abbreviations

DFI: Diabetic foot infection
DFU: Diabetic foot ulcer
DM: Diabetes Mellitus
DPN: Diabetic peripheral neuropathy
MRI: Magnetic resonance imaging
T1DM: Type 1 Diabetes Mellitus
T2DM: Type 2 Diabetes Mellitus
